# The association of dialysis adequacy, body mass index, and mortality among hemodialysis patients

**DOI:** 10.1186/s12882-019-1570-0

**Published:** 2019-10-22

**Authors:** Woong-pyo Hong, Yu-Ji Lee

**Affiliations:** Division of Nephrology, Department of Medicine, Samsung Changwon Hospital, Sungkyunkwan University School of Medicine, 158, Paryong-ro, Masanhoewon-gu, 51353 Changwon, Republic of Korea

**Keywords:** Body mass index, Dialysis adequacy, Hemodialysis, Mortality

## Abstract

**Background:**

Although hemodialysis (HD) adequacy, single-pool Kt/V_urea_ (spKt/V), is inversely correlated with body size, each is known to affect patient survival in the same direction. Therefore, we sought to examine the relationship between HD adequacy and mortality according to body mass index (BMI) in HD patients and explore a combination effect of BMI and HD adequacy on mortality risk.

**Methods:**

We retrospectively reviewed patient data from the Korean Society of Nephrology registry, a nationwide database of medical records of HD patients, from January 2001 to June 2017. We included patients ≥18 years old who were receiving maintenance HD. Patients were categorized into three groups according to baseline BMI (< 20 (low), 20 to < 23 (normal), and ≥ 23 (high) kg/m^2^). Baseline spKt/V was divided into six categories.

**Results:**

Among 18,242 patients on HD, the median follow-up duration was 5.2 (IQR, 1.9–8.9) years. Cox regression analysis showed that, compared to the reference (spKt/V 1.2–1.4), lower and higher baseline spKt/V were associated with greater and lower risks for all-cause mortality, respectively. However, among patients with high BMI (*n* = 5588), the association between higher spKt/V and lower all-cause mortality was attenuated in all adjusted models (*P*_interaction_ < 0.001). Compared to patients with normal BMI and spKt/V within the target range (1.2–1.4), those with low BMI had a higher risk for all-cause mortality at all spKt/V levels. However, the gap in mortality risk became narrower for higher values of spKt/V. Compared to patients with normal BMI and spKt/V in the target range, those with high BMI and spKt/V < 1.2 were not at increased risk for mortality despite low dialysis adequacy.

**Conclusions:**

The association between spKt/V and mortality in HD patients may be modified by BMI.

## Background

Despite constant efforts to improve the survival of hemodialysis (HD) patients, which have produced tangible results, their mortality rate remains high [1, 2]. Several factors affect survival in HD patients, with dialysis adequacy representing one of the most important and modifiable factors [3, 4]. Single-pool Kt/V_urea_ (spKt/V), which is urea clearance multiplied by duration of treatment session and normalized for urea distribution volume, is universally employed as a measure of delivered dialysis dose. Among HD patients, values of spKt/V less than 1.2 are associated with increased mortality [5, 6]. Therefore, the National Kidney Foundation-Kidney Dialysis Outcome Quality Initiative (NKF-KDOQI) clinical practice guidelines for dialysis adequacy currently recommend a target spKt/V of 1.4 per session to obtain a minimum delivered spKt/V of 1.2 for patients receiving HD on a thrice-weekly schedule [7].

spKt/V is inversely correlated with body size because it is normalized for volume of urea distribution. As a result, patients with higher body mass index (BMI) are more likely to have lower Kt/V, which is associated with increased mortality, and vice versa [8]. Among the general population, obesity is associated with increased mortality and morbidity [9]. However, a higher BMI, taken as an indicator of nutritional status, is paradoxically associated with increased survival in HD patients [10, 11]. Therefore, while Kt/V and BMI are negatively correlated, they may affect patient survival in the same direction.

Given that Kt/V tends to overestimate and underestimate delivered dialysis among smaller and larger patients, respectively, we sought to examine whether the association of HD adequacy with mortality differs among patients according to BMI category. In addition, we examined a combination effect of BMI and HD adequacy on mortality risk.

## Methods

### Study population

We retrospectively extracted and examined patient data from the Korean Society of Nephrology registry, a nationwide dataset of medical records of patients with end-stage renal disease (ESRD), from January 1, 2001 to June 31, 2017. Patients 18 years or older receiving maintenance HD on a twice- or thrice-weekly schedule were included in the study cohort. We excluded patients who did not have data on baseline spKt/V and BMI. The time of registry enrollment was set as the time of study enrollment. Patients were followed until death, loss to follow-up, transplantation, or end of study enrollment period (June 2017).

The study was approved by the Institutional Review Committees of Samsung Changwon Hospital, Sungkyunkwan University School of Medicine, who waived the requirement for informed consent.

### Demographic and laboratory measurements

Information on age, sex, ESRD reason (diabetes, hypertension, glomerulonephritis, or others), comorbidities (diabetes, hypertension, and cardiovascular disease), dialysis vintage (from initiation of HD to study enrollment), body weight, height, dialysis adequacy (spKt/V), dialysis prescription, systolic and diastolic blood pressure (SBP and DBP), normalized protein catabolic rate (nPCR), and hemoglobin level were recorded. Demographic data were collected at the time of study enrollment. Clinical and laboratory data within 3 months from study enrollment were collected. Repeated measures of variables for each patient for the 3 months were averaged and used as baseline data.

The spKt/V was calculated as follows: spKt/V = − ln(R −  0.008 × t) + (4 − 3.5 ×R) × UF/W, where R is the ratio of pre- to post-HD concentration of blood urea nitrogen (BUN), t is dialysis session length (in hours), UF is amount of ultrafiltration (L) during the given HD session, and W is post-HD weight (kg). BMI was calculated using post-HD body weight in kilograms divided by square of height in meters. Residual kidney function (RKF) expressed in mL/min was calculated as follows: collected urine volume × [urine creatinine/serum creatinine + urine urea/(0.25 × post-HD BUN + 0.75 × midweek pre-HD BUN)]/(2 × urine collection time × 60). Urea reduction ratio (URR) was calculated by dividing the difference of pre- and post-HD BUN by pre-HD BUN and multiplying by 100 (%).

### Statistical analyses

Patients were categorized into three groups according to baseline BMI as follows: < 20 (low), 20 to < 23 (normal), and ≥ 23 (high) kg/m^2^. Baseline characteristics were described across these BMI groups, and trends across groups were evaluated using linear regression analysis or non-parametric trend tests as appropriate. Baseline spKt/V was divided into six categories as follows: < 1.0, 1.0 to < 1.2, 1.2 to < 1.4, 1.4 to < 1.6, 1.6 to < 1.8, and ≥ 1.8. The association of spKt/V with all-cause mortality was examined by Cox proportional hazards regression models using the spKt/V category of 1.2 to < 1.4 as the reference group. We employed hierarchical adjustment with three models as follows: (1) model 1 included only baseline spKt/V or URR; (2) model 2 included the same variables as model 1 as well as case mix variables of age, sex, dialysis vintage, ESRD etiology (diabetes, hypertension, glomerulonephritis, or others), and comorbidities (diabetes, hypertension, and cardiovascular disease); and (3) model 3 included the same variables as model 2 as well as session time, SBP, DBP, presence of RKF (RKF ≥1 mL/min), hemoglobin, and nPCR. The association of spKt/V with all-cause mortality according to BMI strata was examined using the methods described above, as well as restricted cubic spline functions with four knots. For sensitivity analyses, we examined the association of all-cause mortality with URR as another index of HD adequacy according to BMI strata. Effect modification on the association of spKt/V with all-cause mortality by BMI was evaluated by adding an interaction term to the case mix-adjusted model (model 2).

Missing baseline covariates were dialysis vintage, session time, RKF, SBP, DBP, hemoglobin, and nPCR. The frequency of missing data was less than 11% for all variables. Baseline missing data were imputed using multiple imputation with 10 imputed datasets and a Cox regression model. All analyses were carried out using STATA version 14.2 (StataCorp LP, College Station, TX, USA).

## Results

### Patient characteristics

**Fig. 1:**
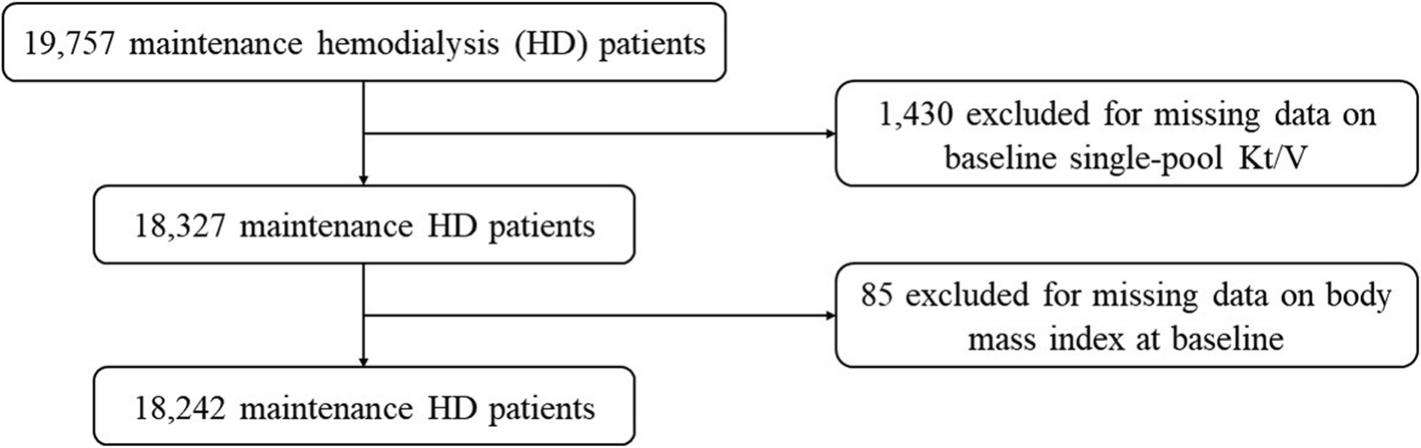
Cohort design

Among 19,757 patients who underwent maintenance HD with twice- or thrice-weekly schedule, we excluded 1430 with missing baseline spKt/V data and 85 patients without a baseline BMI measurement. The final study cohort consisted of 18,242 maintenance HD patients (Fig. 1).

The baseline characteristics of the patients according to BMI category are shown in Table 1. The mean age (± SD) was 59 ± 14 years, 59% were male, 43% had ESRD due to diabetic nephropathy, and the mean baseline spKt/V value was 1.4 ± 0.3. Patients with higher BMI were more likely to be male, have diabetes and RKF, and tended to have lower spKt/V and URR values but higher hemoglobin and nPCR levels.

**Table 1:** Baseline characteristics of 18,242 hemodialysis patients stratified by baseline body mass index

Variables	Body mass index, kg/m^2^				
	Total	< 20	20 to < 23	≥23	*P* value
N (%)	18,242	5681 (31)	6973 (38)	5588 (31)	
Age, years	59 ± 14	58 ± 16	59 ± 14	59 ± 13	0.383
Male, %	59	52	63	61	< 0.001
Vintage, year	1.5 (0.5–4.1)	1.9 (0.5–5.2)	1.5 (0.5–4.2)	1.2 (0.4–3.3)	< 0.001
ESRD reason, %					
Diabetes	43	37	43	49	< 0.001
Hypertension	19	18	19	20	0.031
Glomerulonephritis	12	15	12	10	< 0.001
Others	26	30	26	21	< 0.001
Comorbidities, %					
Diabetes	43	37	43	49	< 0.001
Hypertension	30	30	30	29	0.088
Cardiovascular disease	8	8	8	8	0.359
Single pool Kt/V	1.4 ± 0.3	1.5 ± 0.4	1.4 ± 0.3	1.4 ± 0.3	< 0.001
Urea reduction ratio, %	69 (64–74)	72 (67–76)	69 (64–74)	67 (63–72)	< 0.001
Treatment time, min	239 ± 11	238 ± 12	239 ± 10	239 ± 11	< 0.001
Predialysis SBP, mmHg	146 ± 20	145 ± 20	146 ± 20	145 ± 19	0.236
Predialysis DBP, mmHg	81 ± 11	82 ± 11	81 ± 11	81 ± 11	< 0.001
Presence of RKF, %	6	5	6	7	< 0.001
Hemoglobin, g/dL	10.1 ± 1.3	9.9 ± 1.3	10.1 ± 1.2	10.2 ± 1.2	< 0.001
nPCR, g/kg/day	1.0 ± 0.8	0.9 ± 0.7	1.0 ± 0.8	1.0 ± 0.8	< 0.001

Values for categorical variables are shown as percentages; values for continuous variables, as mean ± standard deviation or median (interquartile range)

*Abbreviations*: *DBP* diastolic blood pressure, *ESRD* end-stage renal disease, *nPCR* normalized protein catabolic rate, *RKF* residual kidney function, *SBP* systolic blood pressure

#### Association of spKt/V and all-cause mortality according to body mass index strata

**Fig. 2:**
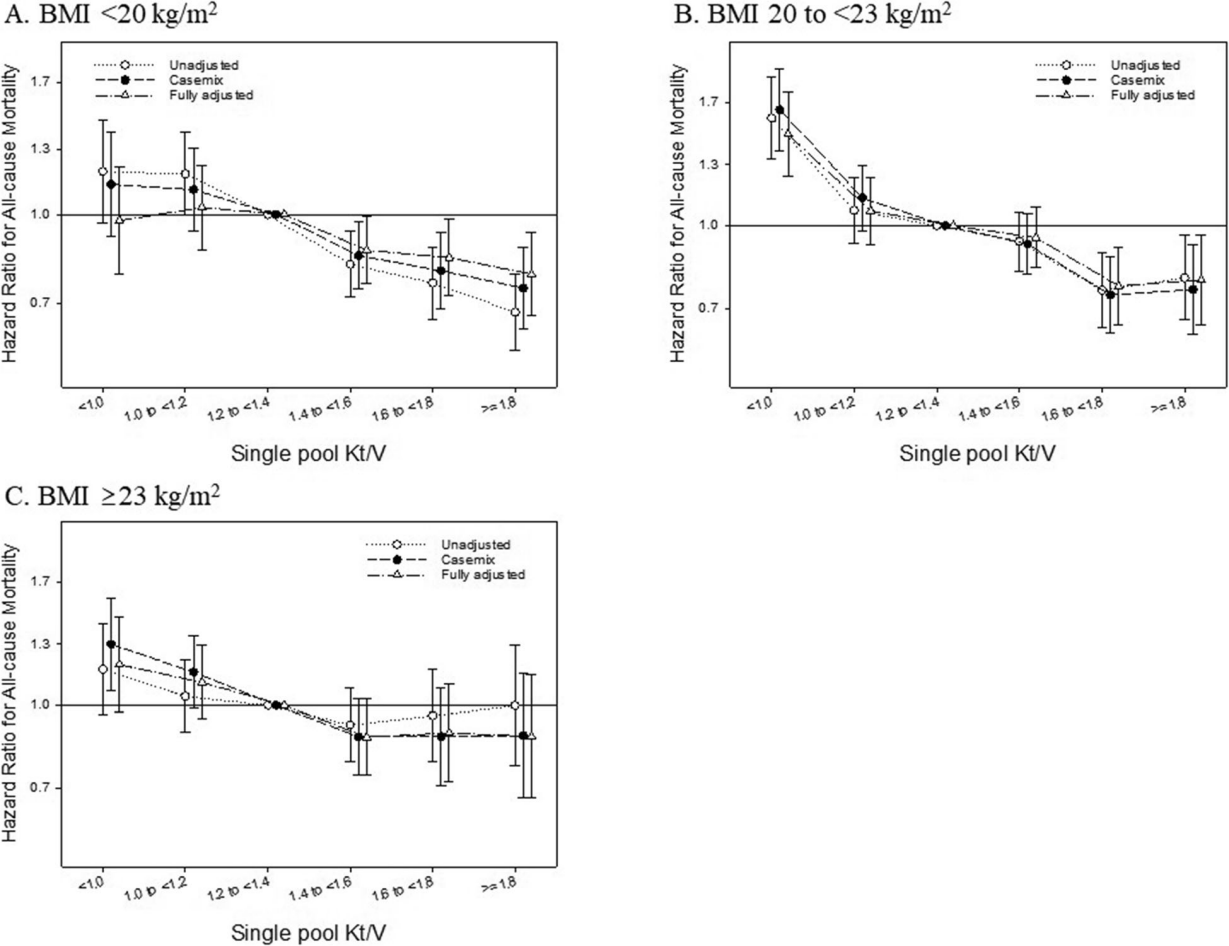
Single-pool Kt/V and hazard ratios (95% confidence intervals) for all-cause mortality stratified by baseline body mass index (BMI) < 20 kg/m^2^, 20 to < 23 kg/m^2^, and ≥ 23 kg/m^2^ (**a**, **b**, and **c**, respectively) among 18,242 hemodialysis patients

Among patients on maintenance HD, the median follow-up duration was 5.2 (IQR, 1.9–8.9) years. During this period, we identified 4824 (44.4 per 1000 pt-yrs) all-cause deaths. In restricted cubic spline models, higher spKt/V and BMI were independently associated with lower risk for all-cause mortality, respectively (see Additional file 1: Figure S1). Cox regression analyses showed that, compared to the reference (spKt/V 1.2 to < 1.4), lower and higher baseline spKt/V values were associated with greater and lesser risks for all-cause mortality, respectively; the case mix-adjusted hazard ratios (HRs) and 95% confidence intervals (CIs) were 1.33 (1.19–1.49), 1.09 (1.00–1.19), 0.93 (0.86–1.01), 0.86 (0.78–0.95), and 0.86 (0.77–0.96) for spKt/V values < 1.0, 1.0 to < 1.2, 1.4 to < 1.6, 1.6 to < 1.8, and ≥ 1.8, respectively (Table 2). We found that the association of baseline spKt/V with all-cause mortality was significantly modified by BMI (*P*_interaction_ < 0.001). Among patients with low (< 20 kg/m^2^) and normal BMI (20 to < 23 kg/m^2^), higher spKt/V was associated with lower all-cause mortality compared to the reference group in all adjusted models (Fig. 2a and b). On the other hand, among patients with high BMI (≥23 kg/m^2^), the association between higher spKt/V and lower all-cause mortality was attenuated in all adjusted models (Fig. 2c). The association between continuous spKt/V level and mortality according to BMI strata was also robust in the restricted cubic spline models (see Additional file 1: Figure S2). These results were consistent even after restricting the analysis to only those patients receiving thrice-weekly HD (*n* = 16,182) (see Additional file 1: Table S1).

**Table 2:** Adjusted hazard ratios for all-cause mortality according to categorized single-pool Kt/V (spKt/V) among 18,242 hemodialysis patients

spKt/V	Model 1		Model 2		Model 3	
	HRs	95% CI	HRs	95% CI	HRs	95% CI
< 1.0	1.3	1.16–1.45	1.33	1.19–1.49	1.17	1.05–131
1.0 to < 1.2	1.05	0.96–1.15	1.09	1.00–1.19	1.03	0.94–1.13
1.2 to < 1.4	Reference		Reference		Reference	
1.4 to < 1.6	0.92	0.85–1.00	0.93	0.86–1.01	0.95	0.87–1.02
1.6 to < 1.8	0.85	0.78–0.94	0.86	0.78–0.95	0.89	0.81–0.98
≥1.8	0.85	0.76–0.94	0.86	0.77–0.96	0.88	0.79–0.99

Model 1: Adjusted for only baseline spKt/V

Model 2: Adjusted for covariates of model 1 and age, sex, dialysis vintage, end-stage renal disease reason (diabetes, hypertension, glomerulonephritis, or others), and comorbidities (diabetes, hypertension, and cardiovascular disease)

Model 3: Adjusted for covariates of model 2 and session time, systolic blood pressure, diastolic blood pressure, presence of residual kidney function (renal urea clearance ≥1 mL/min), hemoglobin, and normalized protein catabolic rate

### All-cause mortality in relation to body mass index and spKt/V

**Fig. 3:**
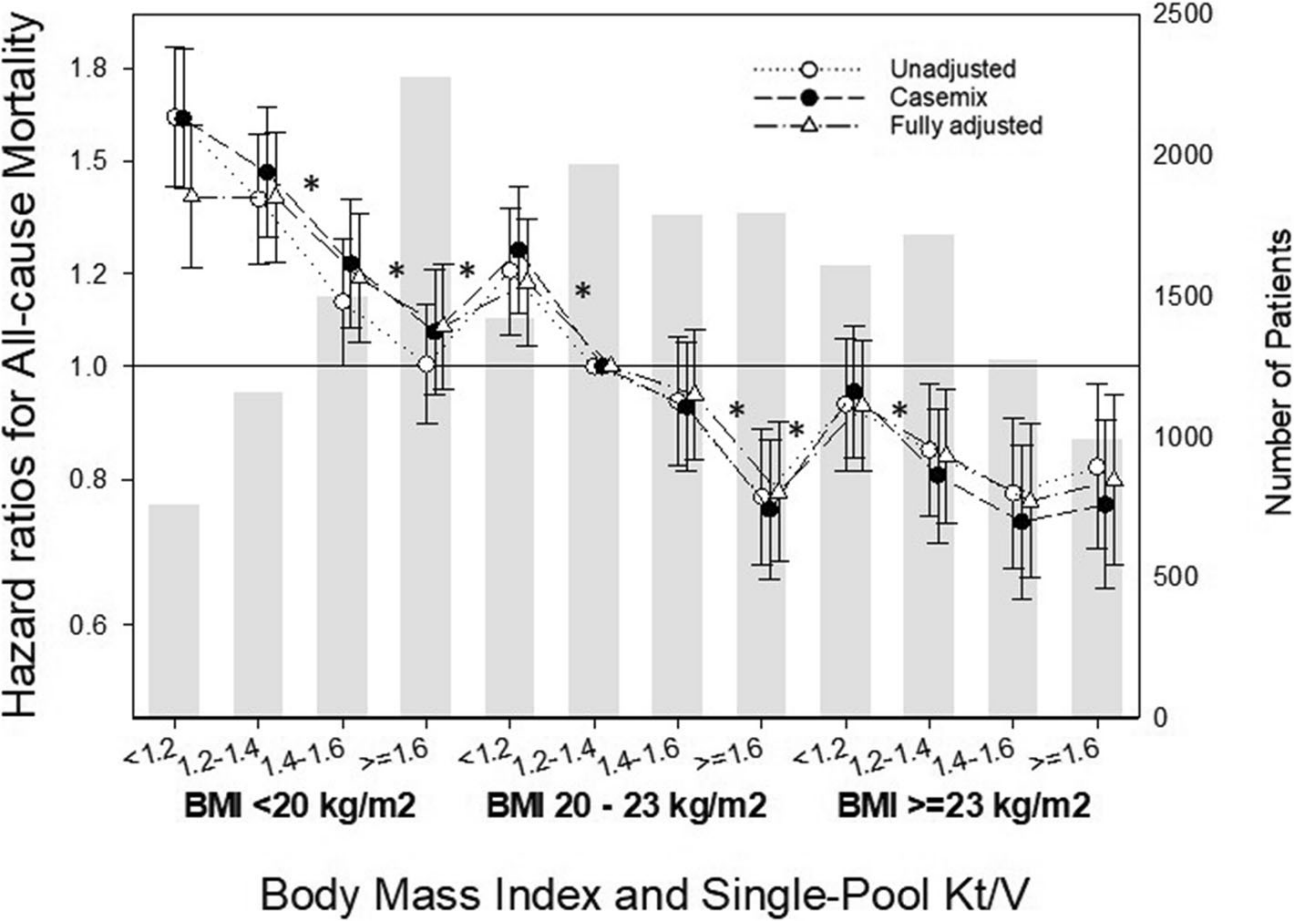
Hazard ratios (95% confidence intervals) for all-cause mortality according to body mass index (BMI) and single-pool Kt/V. ^*^P < 0.05 compared with the previous level

Compared to patients with normal BMI (20 to < 23 kg/m^2^) and spKt/V within the target range of 1.2 to < 1.4, those with low BMI (< 20 kg/m^2^) had higher risk for all-cause mortality for all spKt/V categories (Fig. 3). However, increasing spKt/V values was associated with gradual narrowing of the gap in mortality risk. Compared to patients with normal BMI and spKt/V within the target range, those with high BMI (≥23 kg/m^2^) and spKt/V < 1.2 did not have increased risk for mortality despite low dialysis adequacy. Moreover, patients with high BMI and spKt/V ≥ 1.2 were at lower risk for all-cause mortality compared to those with normal BMI and spKt/V within the target range. However, increasing the spKt/V beyond that did not afford any additional survival benefit compared to the next lower spKt/V category among HD patients with high BMI.

### Sensitivity analysis by urea reduction ratio as an index of hemodialysis adequacy

**Fig. 4:**
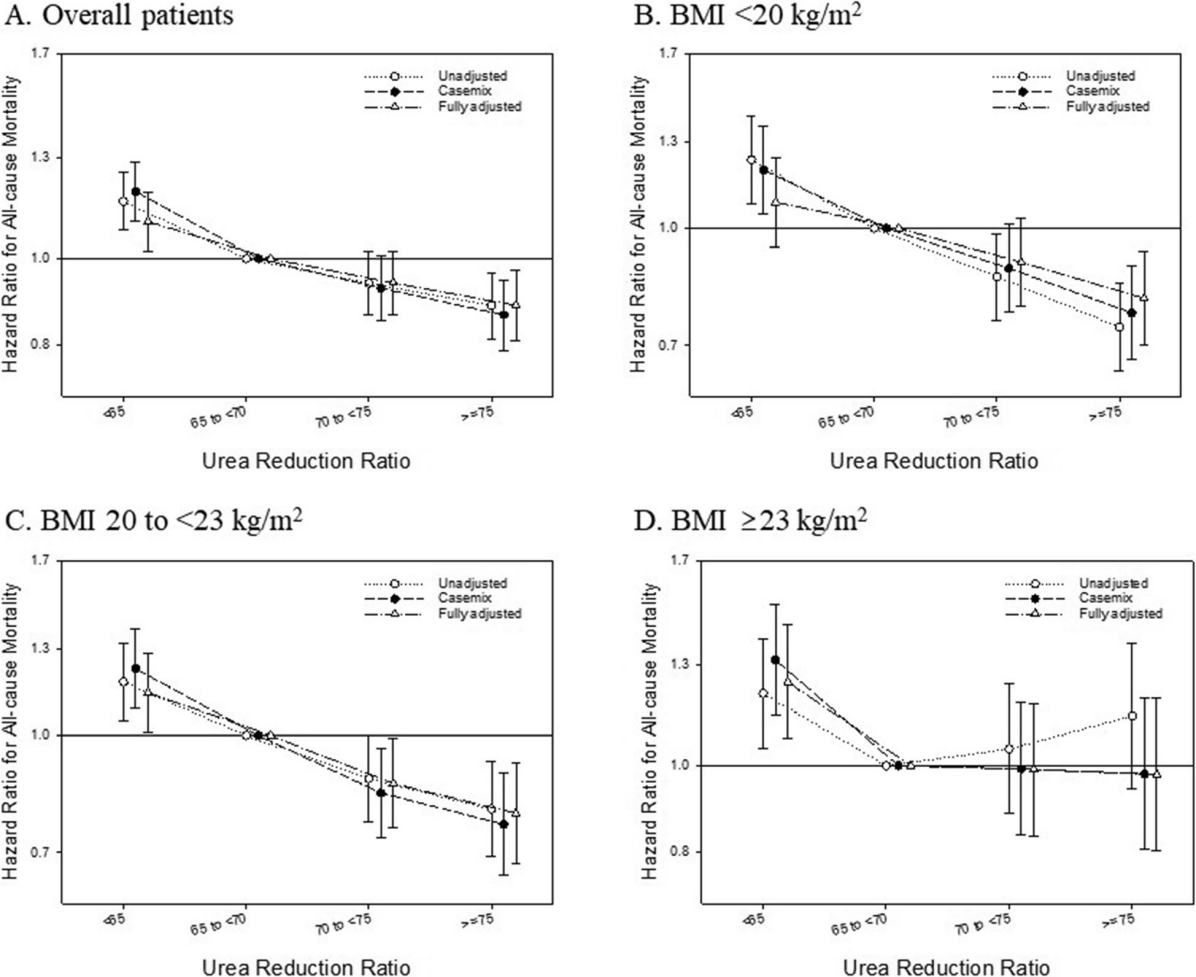
Urea reduction ratio as an alternative index of dialysis adequacy and associated hazard ratio (95% confidence interval) for all-cause mortality in overall cohort (**a**) and patients with body mass index (BMI) <20 kg/m^2^, 20 to <23 kg/m^2^, and ≥23 kg/m^2^ (**b**, **c**, and **d**, respectively)

The median (IQR) baseline URR in our cohort was 69 (64–74)%. The association of URR with all-cause mortality was also modified by BMI, which was consistent with the finding of spKt/V (Fig. 4). Among patients with low (< 20 kg/m^2^) or normal BMI (20 to < 23 kg/m^2^), higher URR was associated with lower risk for all-cause mortality compared to the reference group (URR 65 to < 70). On the other hand, the association of higher URR with lower mortality was attenuated among patients with high BMI (≥23 kg/m^2^).

## Discussion

The results of the present study suggest that the effect of dialysis adequacy on mortality among maintenance HD patients may be modified by BMI. In the high BMI group, the association between dialysis adequacy and mortality was attenuated. Moreover, we identified a combination effect of BMI and spKt/V on mortality. Specifically, patients with low BMI had higher mortality risk even when they had higher spKt/V compared to those with normal BMI and spKt/V within the target range. In contrast, patients with high BMI tended to have lower mortality risk at any level of spKt/V compared to those with normal BMI and spKt/V within the target range, but there was no additional benefit on mortality of spKt/V value higher than the target range.

Current clinical practice guidelines recommend a target spKt/V of 1.2 to 1.4 per session [7]. According to the results of the Hemodialysis (HEMO) study, doses of dialysis beyond the recommended target dose appear to have minimal benefit on survival [12]. However, the association between dialysis dose and survival may not be uniform across all patients [13]. In our study, there was a significant positive association between higher spKt/V above the target and improved survival among HD patients with BMI < 23 (low and normal BMI). In contrast, survival in the high BMI group was not significantly improved at higher doses of dialysis. These results are similar to findings described by Port et al., who identified a significant association between higher URR and improved survival in small and medium BMI groups receiving HD, with the exception of the highest BMI group, in which the mortality risk plateaued in the highest URR category [14]. In addition, Wang et al. reported that increasing dialysis dose in overweight HD patients does not lead to improved health-related quality of life [15].

The differences between the low and high BMI groups observed in our study may be explained in several ways. First, malnutrition-inflammation complex syndrome and protein energy wasting likely account for the relationship between lower BMI and greater mortality among dialysis patients [10, 16]. Indeed, improvement of dialysis adequacy has been associated with better nutritional status as assessed by nPCR and serum albumin, which together are linked to greater survival [17]. However, high BMI is also likely indicative of good nutritional condition and is less likely to be affected by the beneficial effects related to higher dialysis adequacy. Post-dialysis urea rebound and rate of urea generation represent a second explanation for the differences observed in our study according to BMI group. Specifically, spKt/V can overestimate the delivered KtV by up to 15–40% due to the post-dialysis urea rebound phenomenon [18, 19]. Smaller patients who have a lower urea distribution volume tend to experience higher post-dialysis urea rebound [20]. Increased spKt/V may also help achieve a more appropriate delivered Kt/V in smaller patients. Furthermore, uremic toxin generation per unit of body mass is greater in patients with low body mass. Therefore, the concentration of uremic toxins in body fluid tends to be greater in small patients, who in turn may have higher requirement for dialysis [21, 22].

By simultaneously considering BMI and spKt/V, our results showed a higher mortality rate in patients with low BMI for any spKt/V compared to patients with normal BMI and spKt/V within the target range. In addition, patients with low BMI benefited from a higher dialysis dose. In contrast, patients with high BMI had lower mortality at all levels of spKt/V compared to patients with normal BMI and spKt/V within the target range, with the difference in effects in patients with spKt/V < 1.2 failing to reach statistical significance. The negative effect of underdialysis in overweight HD patients might have been counteracted by high BMI itself. This possibility is partially supported by the results of Owen et al., who reported that lower URR is not associated with increased mortality in African Americans with higher BMI and better nutrition status compared to Caucasians [13]. Therefore, our results indicate that the impact on mortality derived from nutritional status by higher BMI may outbalance the impact of given HD dose on mortality.

Our study has several potential limitations that should be addressed. First, although we adjusted for several confounding factors to examine the association between spKt/V and mortality modified by BMI, we were unable to exclude the presence of residual confounding factors due to the observational nature of the study. In particular, serum albumin or inflammatory markers associated with malnutrition-inflammation complex syndrome and mortality were not included in the analysis because of missing data. In addition, body weight and dialysis adequacy may change over time. Therefore, we were unable to prove causation. Second, we did not consider RKF when calculating spKt/V. HD patients with RKF may have better survival rates despite low spKt/V; however, such a case would likely have biased our results toward the null.

## Conclusions

Our study shows that the association between dialysis adequacy assessed by spKt/V and mortality in HD patients may be modified by BMI. Patients with low or normal BMI experienced a decreased risk of mortality by increasing spKt/V above target, while those with high BMI gained no additional benefit from increasing spKt/V above the target. This study highlights the need for individualized spKt/V targets in HD patients and a flexible approach to treatment targets during patient-centered care. Further clinical trials are needed to confirm our results and to elucidate the causative nature of the associations observed in this study.

## Supplementary information

**Additional file 1:** **Table S1.** Case mix-adjusted hazard ratios for all-cause mortality according to categorized single-pool Kt/V (spKt/V) among 16,182 thrice-weekly hemodialysis patients stratified by body mass index. **Figure S1.** Case mix-adjusted restricted cubic spline model showing hazard ratios for all-cause mortality according to baseline single-pool Kt/V level and body mass index. **Figure S2.** Case mix-adjusted restricted cubic spline model showing hazard ratios for all-cause mortality by baseline single-pool Kt/V level according to body mass index (BMI).

## Data Availability

The data that support the findings of this study were used under license for the current study from the Korean Society of Nephrology and are not publicly available. Data are, however, available from the authors upon reasonable request and with permission of the Korean Society of Nephrology.

## Abbreviations

BMI: Body mass index
BUN: Blood urea nitrogen
DBP: Diastolic blood pressure
ESRD: End-stage renal disease
HD: Hemodialysis
IQR: Interquartile range
NKF-KDOQI: National Kidney Foundation-Kidney Dialysis Outcome Quality Initiative
nPCR: normalized protein catabolic rate
RKF: Residual kidney function
SBP: Systolic blood pressure
spKt/V: single-pool Kt/V_urea_
UF: Ultrafiltration; URR, urea reduction ratio
