# Supplementary material for: The association of dialysis adequacy, body mass index, and mortality among hemodialysis patients
Source: BMC Nephrol. 2019 Oct 22;20:382. doi: 10.1186/s12882-019-1570-0 (PMC6805311; doi:10.1186/s12882-019-1570-0)
Supplement: Supplementary file 1 — Additional file 1: Table S1. Case mix-adjusted hazard ratios for all-cause mortality according to categorized single-pool Kt/V (spKt/V) among 16,182 thrice-weekly hemodialysis patients stratified by body mass index. Figure S1. Case mix-adjusted restricted cubic spline model showing hazard ratios for all-cause mortality according to baseline single-pool Kt/V level and body mass index. Figure S2. Case mix-adjusted restricted cubic spline model showing hazard ratios for all-cause mortality by baseline single-pool Kt/V level according to body mass index (BMI). [file 12882_2019_1570_MOESM1_ESM.docx]

**Additional file**

Table S1. Case mix-adjusted hazard ratios for all-cause mortality according to categorized single-pool Kt/V (spKt/V) among 16,182 thrice-weekly hemodialysis patients stratified by body mass index.

| spKt/V | BMI <20 kg/m2 | |  | BMI 20 to <23 kg/m2 | |  | BMI ≥23 kg/m2 | |
| --- | --- | --- | --- | --- | --- | --- | --- | --- |
|  | HRs | 95% CI |  | HRs | 95% CI |  | HRs | 95% CI |
| <1.0 | 1.09 | 0.87-1.37 |  | 1.74 | 1.45-2.10 |  | 1.30 | 1.06-1.59 |
| 1.0 to <1.2 | 1.16 | 0.97-1.39 |  | 1.20 | 1.03-1.40 |  | 1.15 | 0.99-1.35 |
| 1.2 to <1.4 | Reference | |  | Reference | |  | Reference | |
| 1.4 to <1.6 | 0.89 | 0.77-1.02 |  | 0.94 | 0.82-1.08 |  | 0.87 | 0.74-1.03 |
| 1.6 to <1.8 | 0.81 | 0.69-0.095 |  | 0.72 | 0.61-0.87 |  | 0.87 | 0.71-1.08 |
| ≥1.8 | 0.75 | 0.63-0.90 |  | 0.78 | 0.63-0.95 |  | 0.88 | 0.67-1.15 |

Case mix-adjusted model included baseline spKt/V, age, sex, dialysis vintage, end-stage renal disease reason (diabetes, hypertension, glomerulonephritis, or others), and comorbidities (diabetes, hypertension, and cardiovascular disease).

Fig. S1. Case mix-adjusted restricted cubic spline model showing hazard ratios for all-cause mortality according to baseline single-pool Kt/V level and body mass index.

Fig. S2. Case mix-adjusted restricted cubic spline model showing hazard ratios for all-cause mortality by baseline single-pool Kt/V level according to body mass index (BMI).

B. BMI 20 to <23 kg/m^2^

A. BMI <20 kg/m^2^

C. BMI ≥23 kg/m^2^
